# On the Donor: Acceptor Features for Poly(3-hexylthiophene): TiO_2_ Quantum Dots Hybrid Materials Obtained via Water Vapor Flow Assisted Sol-Gel Growth

**DOI:** 10.3390/polym15071706

**Published:** 2023-03-29

**Authors:** Dominique Mombrú, Mariano Romero, Ricardo Faccio, Alvaro W. Mombrú

**Affiliations:** Centro NanoMat & Área Física, Departamento de Experimentación y Teoría de la Estructura de la Materia y sus Aplicaciones (DETEMA), Facultad de Química, Universidad de la República, Montevideo C.P. 11800, Uruguay

**Keywords:** poly(3-hexylthiophene), TiO_2_ quantum dots, sol-gel, hybrid materials, donor–acceptor, DFT calculations

## Abstract

Here, we present a novel methodology for the preparation of P3HT:TiO_2_ quantum dots hybrid materials via water vapor flow-assisted sol-gel growth focusing on the structural, optical and electrical property characterization complemented with first-principles calculations as a promising donor–acceptor system for polymer and hybrid solar cells. X-ray diffraction and UV-Vis spectroscopy analyses suggest that the increasing concentration of TiO_2_ quantum dots leads to the formation of higher amounts of amorphous regions while the crystalline regions exhibited interesting aspect ratio modifications for the P3HT polymer. Raman spectra evidenced the formation of charge carriers in the P3HT with increasing TiO_2_ quantum dots content and the P3HT:TiO_2_ 50:50 weight ratio resulted in the best composition for optimizing the bulk electronic conductivity, as evidenced by impedance spectroscopy studies. Our DFT calculations performed for a simplified model of the P3HT:TiO_2_ interface revealed that there is an important contribution of the thiophene carbon atoms states in the conduction band at the Fermi level. Finally, our DFT calculations also reveal an evident gain of electron density at the TiO_2_ (101) surface while the thiophene rings showed a loss of the electron density, thus confirming that the P3HT:TiO_2_ junction acts as a good donor–acceptor system. In our opinion, these results not only present a novel methodology for the preparation of P3HT:TiO_2_ quantum dots hybrid materials but also reveal some key aspects to guide the more rational design of polymer and hybrid solar cells.

## 1. Introduction

Recently, there is a growing interest in the preparation of donor–acceptor polymer composites triggered by their use in active layers materials for polymer solar cells [1,2,3]. While the donor material is typically a semiconducting polymer such as thiophene-based conjugated polymers, one of the most popular acceptor materials is [6,6]-phenyl-(C71 or C61)-butyric acid methyl ester (PC61BM or PC71BM) fullerene [4,5]. These fullerene-derived acceptor materials are well known for their high electron affinity and mobility, but also, low absorption in the visible spectra and a high cost of fabrication, making them nonideal candidates for technological applications. For this reason, in the past few years, non-fullerene acceptors have been studied to obtain higher light absorption and lower costs of fabrication [6,7,8,9,10]. In the search for non-fullerene acceptors, important breakthroughs and recent progress have been achieved in the development of polymer donor–polymer acceptor (all-polymer) bulk heterojunction solar cells [11]. However, hybrid metal oxide–polymer solar cells also represent an emerging technology that holds the advantage of pronounced difference in dielectric constants of electron donor and acceptor compounds, controllable phase separation, and chemical stability compared to conventional organic photovoltaic [12]. The preparation of polymer solar cells based on poly(3-hexylthiophene) (P3HT) donor using inorganic nanoparticles acceptors such as the low-cost and nontoxic titanium oxide (TiO_2_) has been already reported [13,14,15]. Other more recent approaches for P3HT:TiO_2_ donor–acceptor materials with TiO_2_ in the form of a mesoporous matrix have been reported to yield slightly higher power conversion efficiency values [16]. The use of small molecules and oligomers as modifiers for P3HT:TiO_2_ hybrid solar cells has also been recently explored yielding an enhancement in their performances [17,18,19]. However, up to now, only a few studies have been reported on fundamental aspects of P3HT:TiO_2_ hybrid materials for solar cell applications, and thus we have poor information to rationally improve their performances. For instance, Leijtens et al. have shown that the mobility of the materials is heavily dependent on the charge carrier density as well as the morphology employing transient absorption spectroscopy combined with time-resolved photo-conductivity measurements [20]. Frischknecht et al. have shown that P3HT:TiO_2_ hybrid solar cells present a relevant dependence of the photocurrent on the incident light wavelength, exhibiting a particularly strong photocurrent enhancement upon UV monochromatic illumination due to the filling of shallow traps that become donor sites with an n-doping effect improving the titania electron mobilities [21]. It has been demonstrated that the use of time-of-flight (TOF) measurements are quite appropriate for observing the effects of the molecular structures, trap states, scattering centers, and dispersivity on hole/electron carrier transport [22,23,24,25,26]. In one of the later reports, bulk heterojunctions are studied in thick (>1 μm) devices showing that electron transport occurs mainly by diffusion in the bulk of the active layer [24]. In addition, other TOF measurement studies have shown highly unbalanced transport for which the hole transport shows trap-free behavior while the electron transport heavily shows trap-limited behavior [26]. However, to the best of our knowledge, there is still a lack of insights into and correlations between the structural, optical and electrical properties of these hybrid materials considering the semi-crystalline nature of P3HT coexisting with the crystalline TiO_2_ nanostructures. For instance, in most studies reported in the literature, hybrid polymer–inorganic nanocomposites are treated as a biphasic system and typically the P3HT polymer phase is treated as full crystalline or full amorphous, and no structural nor physical chemistry in-depth characterization of the material is provided. There are almost no reports studying these systems, particularly their electronic properties related to their donor–acceptor interface from a first-principles approach probably due to their large computational cost. In this manuscript, we present a novel methodology for the preparation of P3HT:TiO_2_ quantum dots hybrid materials via water vapor flow-assisted sol-gel growth. In addition, we focus on the structural, optical and electrical properties characterization complemented with first-principles calculations revealing some key aspects of their donor–acceptor interactions that can be very useful to guide the more rational design of polymer and hybrid solar cells among other applications.

## 2. Materials and Methods

### 2.1. Experimental Section

The preparation of P3HT:TiO_2_ nanocomposites was based on the sol-gel synthesis via water vapor flow diffusion, as it was previously reported for other polymers [27,28,29]. In total, 0.3 g of regioregular poly(3-hexylthiophene-2,5-diyl) (P3HT) polymer, purchased from Sigma-Aldrich with a M_w_ of 50,000–100,000 and a regioregularity above 90%, were suspended in 50 mL of tetrahydrofuran (THF) and kept stirred at T = 70 °C. Then, 1 mL of deionized water was added slowly dropwise. The corresponding amount of titanium tetrapropoxide (TTP) was added to the P3HT suspension and kept stirred at T = 70 °C until dryness. The resulting powder was exposed to deionized water vapor at T = 80 °C with a vapor flow of ~1 mL/min for 16 h. All samples were dried at T = 70 °C under vacuum for 7 h in order to eliminate residual water and propanol generated from the hydrolysis process. Finally, the samples were pressed in the form of pellets with a 1.2 cm^2^ diameter and a 0.1 cm thickness. The samples that corresponded to 30%, 50% and 70% of the weight fraction of TiO_2_ quantum dots were named ×30, ×50 and ×70, respectively.

### 2.2. Characterization of Samples

X-ray diffraction powder (XRD) was performed using a Rigaku Ultima IV diffractometer with CuKα radiation in a Bragg–Brentano configuration, in the 2θ = 2.00–80.00° range, using steps of 0.02°, with 10 s integration time per step. MicroRaman imaging and corresponding spectra for all samples were collected using WITec Alpha 300-RA equipment, working with an excitation laser of 785 nm wavelength and the laser power below ~10 mW to avoid polymer decomposition. Solid-state UV-Vis reflectance measurements were performed utilizing a UV-vis spectrophotometer Shimadzu UV-2600 with an integrating sphere in the 1400–220 nm range. The AC impedance spectroscopy analysis was performed using a Gamry Reference 3000 impedance analyzer with the deposition of silver electrodes on both sides of the samples. The applied AC voltage amplitude was 100 mV in the 0.1 Hz–1 MHz frequency range with applied DC voltages in the V_DC_ = 0–3 V range using a 0.5 V step.

### 2.3. Computational Section

The computational calculations were performed using Density Functional Theory (DFT) [30,31] using the VASP code (Vienna ab initio simulation package) [32,33,34,35]. Pseudopotentials were applied with a plane-wave basis set with a projector augmented wave (PAW) method [36,37] using a generalized gradient approximation (GGA) as the exchange–correlation function according to Perdew-Burke-Ernzerhof (PBE) [38]. The employed pseudopotentials correspond to the following configurations: 3s^2^ 3p^6^ 3d^2^ 4s^2^ for titanium, 3s^2^ 3p^4^ for sulfur, 2s^2^ 2p^4^ for oxygen, 2s^2^ 2p^2^ for carbon and 1s^1^ for hydrogen atoms. A 4 × 4 × 1 k-point mesh was set for the full Brillouin Zone (vacuum space along c-axis), a 400 eV energy cutoff was utilized to expand the Kohn–Sham orbitals into plane wave basis sets, and dipole corrections were applied along the direction perpendicular to the surface. The structures were then optimized until the forces in all the atoms were lower than a 0.01 eV/Å tolerance value. We simulated the adsorption of poly(3-hexylthiophene-2,5-diyl) (P3HT) on TiO_2_ anatase (101) surface after the previous optimization of isolated counterparts. We used a 3-hexylthiophene trimer, named 3M, with the composition C_15_S_3_H_14_ (as a simplified model of P3HT) and a slab model of anatase TiO_2_ (101) as it is the most favorable surface in terms of surface energy [39] consisting of a unit cell with a = 10.5 Å, b = 14.9 Å and c = 25.2 Å, having a vacuum space of ~15 Å aligned along the c-axis. After achieving the optimization of the isolated 3M molecule and TiO_2_ surface, we proceeded to optimize the 3M adsorption on TiO_2_ anatase (101) surface, allowing all the atomic positions to relax and optimize to obtain the joint donor–acceptor system named 3M:TiO_2_.

## 3. Results

**Figure 1:**
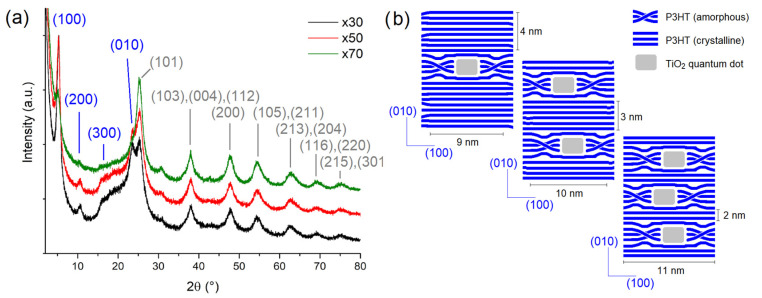
(**a**) X-ray diffraction patterns and (**b**) schematization of most relevant structural features for ×30, ×50 and ×70 nanocomposites.

X-ray diffraction patterns collected for ×30, ×50 and ×70 samples are shown in Figure 1a. Typical XRD profiles for TiO_2_ anatase quantum dots are present in all XRD measurements with broad peaks at 2θ  =  25.4, 38.1, 47.9, 54.6, 62.8, 69.4 and 75.3°, assigned as (101), (103)(004)(112), (200), (105)(211), (213)(204), (116)(220) and (215)(301) planes, respectively [40]. Typical diffraction peaks corresponding to P3HT polymer are detected at 2θ~5.6°, 10.8° and 16.5° ascribed with (100), (200) and (300) Miller planes, assigned with a monoclinic structure with a P2_1_/c space group [41,42]. A broad diffraction peak associated with the amorphous region of P3HT is observed at 2θ~20° according to both experimental and theoretical studies reported in the literature [42,43,44,45,46].

However, particularly ×30 and ×50 samples exhibit a well-defined crystalline peak at 2θ~23.6°, which is associated with the (010) plane of the P3HT crystalline region [42,43,44,45,46].

**Figure 2:**
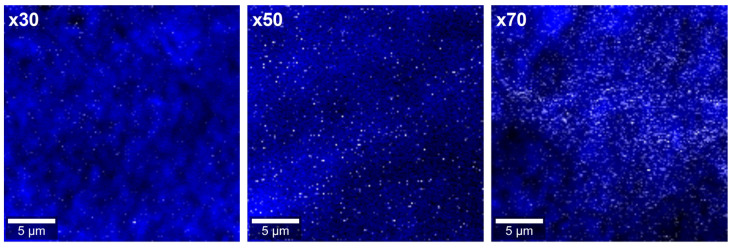
microRaman imaging for ×30, ×50 and ×70 nanocompostites.

As expected, those peaks associated with P3HT become less notorious when the amount of TiO_2_ is higher, becoming practically undetectable for the sample of ×70. To make a quantitative approach, we use the Bragg equation to estimate the most relevant d-spacing distances and we use the Scherrer equation to estimate the most relevant crystalline domain sizes. For this purpose, we perform a Lorentzian deconvolution of selected diffraction peaks; i.e., the (101) plane for TiO_2_ in its anatase polymorph and the (100) and (010) planes for P3HT crystalline regions, as depicted in Figure S1. First, no drastic shifting was evidenced for the d-spacing for the (101) plane for TiO_2_ from d = 0.351 nm nor for the mean crystallite size from D = 4.7–5.2 nm for all compositions. Then, the (100) plane for P3HT associated with the in-plane thiophene-to-thiophene distances showed an increase in its corresponding d-spacing from d = 1.66 to 1.70 nm with increasing TiO_2_-QDs concentration from ×30 to ×70. However, the (010) plane for P3HT associated with the out-of-plane thiophene-to-thiophene distances showed no drastic modifications of its corresponding d-spacing d = 0.377 nm with increasing TiO_2_-QDs concentration. Interestingly, we evidenced that the mean crystallite sizes associated with (100) and (010) planes for P3HT crystalline regions exhibited opposite trends with increasing TiO_2_ quantum dots content. The P3HT mean crystallite size showed an increment from 9.5 to 11.1 nm considering the (100) plane but a decrease from 4.1 to 2.2 nm considering the (010) plane with increasing TiO_2_-QDs concentration from ×30 to ×70, as schematized in Figure 1b. MicroRaman imaging for ×30, ×50 and ×70 nanocompostites are shown in Figure 2. P3HT-rich and TiO_2_-rich regions were defined using the characteristic vibrational modes of P3HT (C=C mode, ~1450 cm^−1^) and TiO_2_ (E_g_ mode, ~140 cm^−1^), colored in blue and white, respectively.

**Figure 3:**
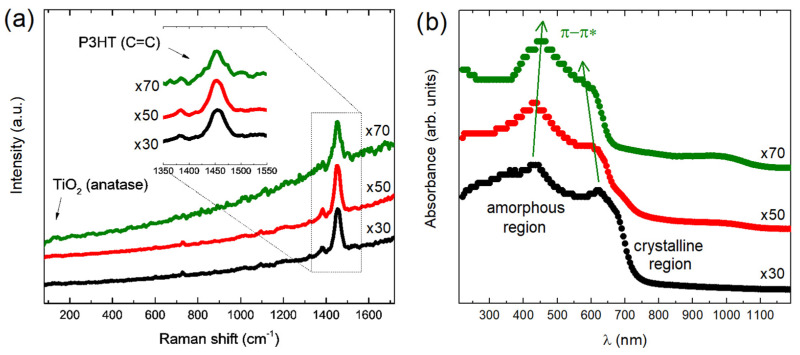
(**a**) microRaman spectra and (**b**) UV-Vis absorbance spectra collected in reflectance mode for ×30, ×50 and ×70 nanocompostites.

**Figure 4:**
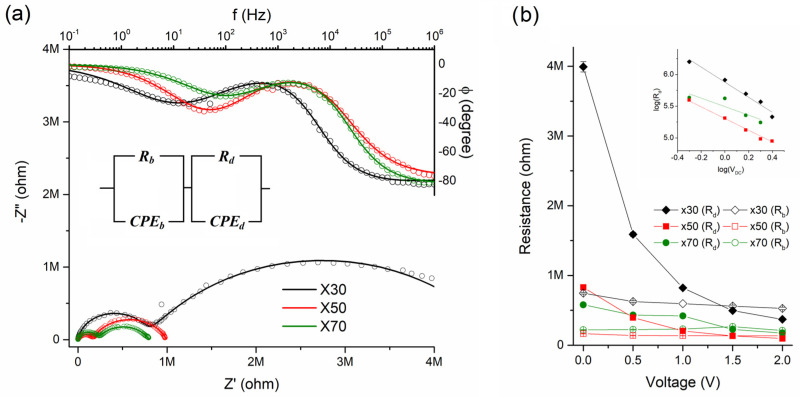
(**a**) Impedance spectroscopy displayed as Bode (**upper** panel) and Nyquist (**lower** panel) and (**b**) Bulk-region (R*_b_*) and depletion-region (R*_d_*) resistances as a function of applied dc voltage (V_DC_) and log(R*_d_*) vs. log(V_DC_) plots (inset) for ×30, ×50 and ×70.

Excellent homogeneity and no drastic segregation were observed for all cases, as evidenced in Figure 2. The averaged microRaman spectra for ×30, ×50 and ×70 nanocomposites are shown in Figure 3a and all of them presented peaks at 1380 cm^−1^ ascribed to the C–C intra-ring stretching mode and 1450 cm^−1^ ascribed to the C=C bond stretching associated with thiophene rings of the P3HT [47]. In the case of ×50 and ×70 samples, a shoulder peak emerges at approximately 1430 cm^−1^, which could be associated with the formation of charge carriers in the thiophene rings of P3HT [48,49]. The UV-Vis spectra for ×30, ×50 and ×70 nanocomposites collected in the reflectance configuration are shown in Figure 3b. According to the literature, crystalline P3HT presented a characteristic peak at ~600–750 nm, ascribed to the π–π* electronic transitions through P3HT chains [50]. It has been already evidenced that highly ordered single crystals of P3HT composed of closely packed π–π stacked fully extended chains exhibit a UV-Vis absorption peak with a maximum at ~670 nm [51]. On the other hand, the P3HT solution spectrum only exhibits a single broad UV-Vis absorption peak at ~455 nm, mainly related to intra-chain states of individual P3HT chains in a flexible random-coil conformation [51]. The P3HT in the solid state with different levels of crystallinity and disorder is usually observed as a sum of both contributions [51]. In our case, we observe a broad peak at ~600–750 nm associated with crystalline regions of P3HT and a peak at ~450 nm associated with larger amounts of amorphous regions in the P3HT [46]. Interestingly, there is a blue shift of the ~600–750 nm peak and a red shift of the ~450 nm peak when the amounts of TiO_2_ increase, i.e., ×50 and ×70, and these shifts are notorious in comparison with isolated P3HT as observed in our previous work [46]. This could suggest that TiO_2_ quantum dots in the composite leads to the reduction in crystalline regions at the expense of the formation of amorphous regions in the polymer [51,52]. Impedance spectra obtained with 100 mV AC amplitude and zero applied DC bias for ×30, ×50 and ×70 are shown in Figure 4a. Bode plots displayed as phase versus frequency plots are shown in the upper panel of Figure 4a and Nyquist plots represented as imaginary (−Z”) versus real impedance (Z’) are shown in the lower panel of Figure 4a. Both Nyquist and Bode plots were best fitted with the circuit model shown in the inset of Figure 4a, characterized by the series combination of two parallel combinations of a resistor (R) and constant phase element (CPE). The two contributions to the electrical transport can be attributed to different zones in the nanocomposites: one corresponding to a bulk zone (R*_b_*-CPE*_b_*) and the other to a depletion zone (R*_d_*-CPE*_d_*) of the polymer nanocomposites, in agreement with previous reports [28,46]. For all cases, R*_b_*-CPE*_b_* contribution is one order of magnitude lower than the R*_d_*-CPE*_d_* contribution corroborating that the depletion region is governing the whole electronic transport in the samples.

The increasing amounts of TiO_2_ quantum dots lead to a drastic decrease in several orders of magnitude in the total resistance of the nanocomposites. The associated total conductivities were calculated using:

*σ_T_* = *l*/*A*(*R_b_* + *R_d_*)

With *l* and *A* being the thickness and effective electrode area of the samples, yielding *σ_T_* = 2.11 × 10^−8^, 1.00 × 10^−7^ and 1.25 × 10^−7^ S·cm^−1^ for X = 30, 50 and 70, respectively. It is important to note that the total conductivity for P3HT has been reported to be ~3 × 10^−7^ S·cm^−1^ [46] while that for TiO_2_-QDs is expected to be well below 10^−12^ S·cm^−1^ [53]. The P3HT:TiO_2_ bulk conductivity can be defined considering only the bulk resistance following:

*σ_b_* = *l*/*A*·*R_b_*

Yielding *σ_b_* = 1.34 × 10^−7^, 6.05 × 10^−7^ and 4.53 × 10^−7^ S·cm^−1^ for ×30, ×50 and ×70, respectively. This is suggesting that the P3HT:TiO_2_ 50:50 weight ratio is the best composition in optimizing the bulk electronic conductivity, which is comparable to that observed for previous studies on PVK:TiO_2_ obtained by the same preparation technique [28]. The increment of charge carriers with increasing TiO_2_ quantum dots concentration can be explained in terms of P3HT:TiO_2_ donor–acceptor interactions as we will address later in the manuscript when discussing our DFT calculations results. However, the optimization of electronic conductivity for the P3HT:TiO_2_ 50:50 weight ratio can be also interpreted in terms of enhanced thiophene–thiophene interactions as a consequence of structural rearrangements of P3HT conducting chains favored somehow by this critical amount of TiO_2_ quantum dots yielding to an optimization of the percolation pathway of the conducting polymer phase embedded in the TiO_2_ insulating matrix. We also performed impedance spectroscopy at different DC voltages from 0–2 V to have more insight into both electronic transport contributions, as shown in Figure S2. Each electronic resistance contribution as a function of applied dc bias (V_DC_) for ×30, ×50 and ×70 is shown in Figure 4b. The bulk region-associated resistances (R*_b_*) showed a slight decrease with almost constant values while the depleted region-associated resistance (R*_d_*) shows a more drastic decrease with increasing DC voltage.

**Figure 5:**
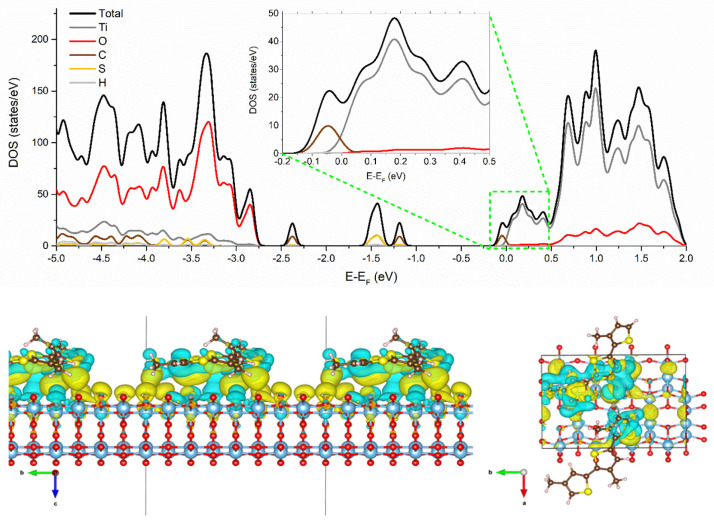
Total and projected density of states (DOS) (**upper** panel) and charge density differences (**lower** panel) for TiO_2_/3M system, where the loss and gain of electron density are represented in blue and yellow, respectively. References for atoms (colors) are carbon (brown), sulfur (yellow), hydrogen (light grey), oxygen (red) and titanium (dark grey).

The larger resistance process related to the dependence of the interface-depleted region resistance (R*_d_*) on the applied DC voltage can be shown by the slope (−*m*) in the log(R*_d_*) vs. log(V_DC_) plots. The *m* values showed *m*~1.2, 1.1 and 0.8 values for ×30, ×50 and ×70, respectively, indicating that the charge carriers exhibit a near ohmic behavior typical of semiconductor material where carriers are generated thermally by the promotion of electrons from the valence band to the conduction band [54]. In our case, the promotion of carriers can be also favored by the presence of donor–acceptor interactions in P3HT:TiO_2_ nanocomposites, as we will discuss later in the manuscript. In the following lines, we will discuss our DFT calculations for our simplified model for the P3HT:TiO_2_ system. First, in order to analyze the adsorption process, the corresponding adsorption energies for all structures were calculated according to the following expression:

∆E = E_3M:TiO2_ − [E_TiO2_ + E_3M_]

where E_3M:TiO2_ is the 3M:TiO_2_ total energy, E_TiO2_ is the total energy for isolated TiO_2_(101) surface and E_3M_ is the isolated 3M trimer total energy. The calculated adsorption energy was E_ads_ = −0.109 eV suggesting a favorable process for 3M:TiO_2_ interaction with respect to their isolated counterparts. In order to discuss the electronic structure of the system, we compute the density of electronic states (DOS) for the 3M:TiO_2_ system as shown in the upper panel of Figure 5. The DOS for the 3M:TiO_2_ system presents a similarity with the typical DOS observed for the isolated TiO_2_ (101) system, characterized by a typical n-type semiconductor behavior with a main contribution of oxygen (O-p states) and titanium (Ti-d states) for the valence and conduction band, respectively [55].

The DOS for the 3M:TiO_2_ system presented characteristic peaks associated with the thiophene oligomer HOMO states mostly below −2.5 eV but also some peaks between −1.1 and −1.7 eV which are mainly associated with π electrons located on the thiophene rings of P3HT oligomer rather than with defect states, as already observed in the literature [56]. In addition, the DOS for the 3M:TiO_2_ system also exhibits an important contribution of the thiophene oligomer LUMO states at the Fermi level, as depicted in the upper panel of Figure 5. This suggests that the thiophene groups are effectively donating electrons toward the TiO_2_ (101) surface. To have more insight into this donor–acceptor interaction, we calculate the charge density difference (Δρ) using the following expression:

∆ρ(r) = ρ(r)_3M:TiO2_ − [ρ(r)_TiO2_ + ρ(r)_3M_]

The charge density difference mapping for the 3M:TiO_2_ system is depicted in the lower panel of Figure 5. There is an evident gain of electron density at the TiO_2_ (101) surface, while the 3M trimer showed a loss of the electron density, thus confirming that the 3M:TiO_2_ junction acts as a good donor–acceptor system. It is important to remark that our DFT calculations refer to a quite simplified model of the real situation in P3HT:TiO_2_ hybrid materials. Nonetheless, it is interesting to point out that our modeling is quite useful to have insights into the P3HT:TiO_2_ interface for TiO_2_ quantum dots most stable surface in contact with P3HT in both amorphous and crystalline forms. This is because the portion of P3HT modeled as the 3M trimer; i.e., an “amorphous” portion of the P3HT chain, is the more probable scenario not only for amorphous P3HT but also for a defectuous grain boundary of crystalline P3HT. However, it is also important to mention that a huge number of other types of spatial configurations, partially for P3HT, can be also present in such a complex organic–inorganic interface. 

## 4. Conclusions

A novel methodology for the preparation of P3HT:TiO_2_ quantum dots hybrid materials via water vapor flow-assisted sol-gel growth has been presented. Our particular focus on the structural, optical and electrical properties characterization revealed interesting features for their potential application in polymer and hybrid solar cells. X-ray diffraction and UV-Vis spectroscopy analyses suggest that the increasing concentration of TiO_2_ quantum dots leads to the formation of higher amounts of amorphous regions in the P3HT polymer. Interestingly, we evidenced that the mean crystallite sizes associated with (100) and (010) planes for P3HT crystalline regions exhibited opposite trends with increasing TiO_2_ quantum dots content. Raman spectra evidenced the formation of charge carriers in the P3HT with increasing TiO_2_ quantum dots content. The P3HT:TiO_2_ bulk conductivity is enhanced for the 50:50 weight ratio suggesting that this is the best composition for optimizing the bulk electronic conductivity. Our DFT calculations performed for a simplified model of the P3HT:TiO_2_ interface revealed that there is an important contribution of the thiophene carbon atoms states in the conduction band at the Fermi level. Finally, our DFT calculations also reveal that there is an evident gain of electron density at the TiO_2_ (101) surface while the thiophene rings showed a loss of the electron density, thus confirming that the P3HT:TiO_2_ junction acts as a good donor–acceptor system. In our opinion, these results not only present a novel methodology for the preparation of P3HT:TiO_2_ quantum dots hybrid materials but also reveal some key aspects to guide the more rational design of polymer and hybrid solar cells.

## Data Availability

The data presented in this study are available on request from the corresponding author.

## Supplementary Materials

The following supporting information can be downloaded at: https://www.mdpi.com/article/10.3390/polym15071706/s1, Figure S1: Selected d-spacing and mean crystalline size as a function of TiO2-QDs concentration; Figure S2: Impedance modulus (upper panel) and phase (middle panel) versus frequency, and Nyquist (lower panel) for (a) ×30, (b) ×50 and (c) ×70 at V_DC_ = 0–3 V.
