# Supplementary material for: On the Donor: Acceptor Features for Poly(3-hexylthiophene): TiO2 Quantum Dots Hybrid Materials Obtained via Water Vapor Flow Assisted Sol-Gel Growth
Source: Polymers (Basel). 2023 Mar 29;15(7):1706. doi: 10.3390/polym15071706 (PMC10096910; doi:10.3390/polym15071706)
Supplement: Supplementary file 1 [file polymers-15-01706-s001.zip › polymers-2272149-supplementary.pdf]

SUPPORTING INFORMATION

On the donor:acceptor features for poly(3-hexylthiophene):TiO_2 quantum dots hybrid materials obtained via water vapor flow assisted sol-gel growth

Dominique Mombrú, Mariano Romero*, Ricardo Faccio* and Álvaro W. Mombrú*.

Centro NanoMat & Área Física, Departamento de Experimentación y Teoría de la Estructura de la Materia y sus Aplicaciones (DETEMA), Facultad de Química, Universidad de la República, Montevideo C.P. 11800, Uruguay.

(*) corresponding authors:

Prof. Dr. Mariano Romero (mromero@fq.edu.uy)

Prof. Dr. Ricardo Faccio (rfaccio@fq.edu.uy)

Prof. Dr. Álvaro Mombrú (amombru@fq.edu.uy)

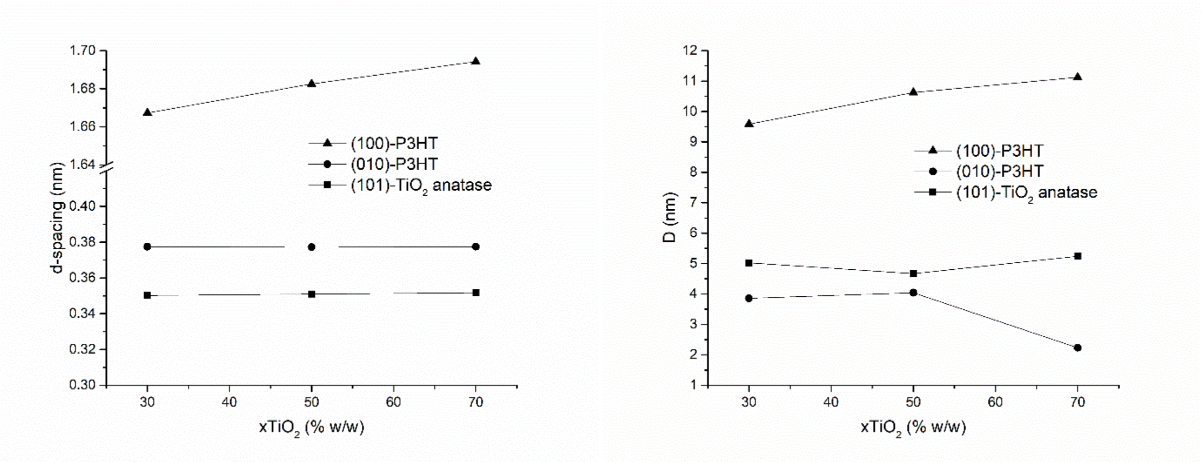

Figure S1. Selected d-spacing and mean crystalline size as a function of TiO_2-QDs concentration.

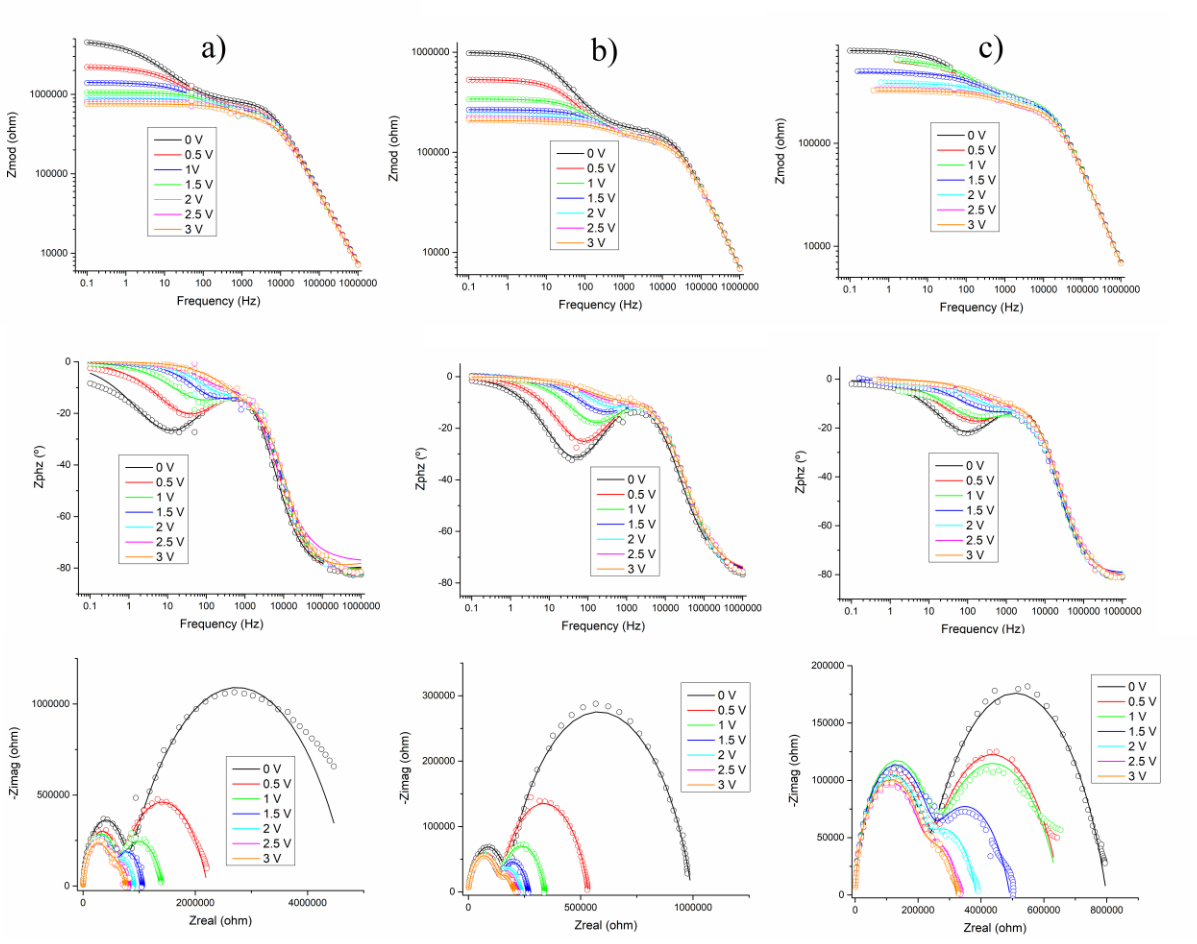

Figure S2. Impedance modulus (upper panel) and phase (middle panel) versus frequency, and Nyquist (lower panel) for a) x30, b) x50 and c) x70 at V_{DC} = 0 – 3 V.
